# Interictal Heart Rate Variability Analysis Reveals Lateralization of Cardiac Autonomic Control in Temporal Lobe Epilepsy

**DOI:** 10.3389/fneur.2020.00842

**Published:** 2020-08-14

**Authors:** Fedele Dono, Giacomo Evangelista, Valerio Frazzini, Catello Vollono, Claudia Carrarini, Mirella Russo, Camilla Ferrante, Vincenzo Di Stefano, Luciano P. Marchionno, Maria V. De Angelis, Massimiliano Faustino, Laura Bonanni, Marco Onofrj, Stefano L. Sensi, Francesca Anzellotti

**Affiliations:** ^1^Department of Neuroscience, Imaging and Clinical Science, “G. D'Annunzio” University of Chieti-Pescara, Chieti, Italy; ^2^AP-HP, Epilepsy Unit, Pitié-Salpêtrière Hospital, and Sorbonne University, Paris, France; ^3^Brain and Spine Institute (INSERM UMRS1127, CNRS UMR7225, Sorbonne Université), Pitié-Salpêtrière Hospital, Paris, France; ^4^Unit of Neurophysiopathology and Sleep Medicine, Department of Geriatrics, Neurosciences and Orthopedics, IRCCS Policlinico Universitario Agostino Gemelli, Catholic University, Rome, Italy; ^5^Department of Biomedicine, Neuroscience and Advanced Diagnostic (BIND), University of Palermo, Palermo, Italy; ^6^Department of Neurology, “SS Annunziata” Hospital, Chieti, Italy; ^7^Department of Cardiology, “SS Annunziata” Hospital, Chieti, Italy; ^8^Center for Advanced Studies and Technology - CAST, “G. D'Annunzio” University of Chieti-Pescara, Chieti, Italy

**Keywords:** temporal lobe epilepsy, interictal epileptic discharges, heart rate variability, autonomic nervous system, cardiovascular risk

## Abstract

**Purpose:** The temporal lobe, a critical hub for cognition, also plays a central role in the regulation of autonomic cardiovascular functions. Lesions in this area are usually associated with abnormalities in the regulation of heart rate (HR) and blood pressure (BP). The analysis of the heart rate variability (HRV) is useful to evaluate the cardiac parasympathetic nervous system activity. This study aims at comparing HRV changes occurring in two groups of patients suffering from Temporal Lobe Epilepsy (TLE). To that aim, we evaluated patients differentiated by the right or left location of the epileptic foci.

**Materials and Methods:** Fifty-two adult patients with a diagnosis of TLE were enrolled. Each patient underwent a 20-min EEG + EKG recording in resting state. According to the localization of epileptic focus, patients were divided into two subgroups: right TLE (R-TLE) and left TLE (L-TLE). HRV parameters were calculated with a short-lasting analysis of EKG recordings. Time-domain and frequency domain-related, as well as non-linear analysis, parameters, were compared between the two groups.

**Results:** Compared to the R-TLE group, L-TLE subjects showed a significant decrease in low frequency (LF) (*p* < 0.01) and low frequency/high-frequency ratio (LF/HF) (*p* < 0.001) as well as increased HF values (*p* < 0.01), a parameter indicative of the presence of an increased cardiac vagal tone. These results were also confirmed in the subgroup analysis that took into account the seizure types, responses to antiepileptic drugs, seizure frequencies, and etiology.

**Conclusions:** The main finding of the study is that, compared to R-TLE, L-TLE is associated with increased cardiac vagal tone. These results indicate that patients with TLE exhibit a lateralized cardiac autonomic control. L-TLE patients may have a lower risk of developing cardiac dysfunctions and less susceptible to develop Sudden Death for Epilepsy (SUDEP).

## Introduction

Temporal lobe epilepsy (TLE) is the most common form of focal epilepsy in adults and accounts for 60% of all epilepsy forms (1). TLE is associated with a large variety of clinical manifestations. A less studied phenomenon associated with TLE and other forms of epilepsy is the simultaneous presence of autonomic imbalance (2–7). However, experimental data indicate that ictal and interictal epileptogenic activity can spread from the temporal lobe and interfere with autonomic functions. Moreover, the temporal lobe plays a central role in the activity of the “Central Autonomic Network” (CAN), a complex system that includes cortical, midbrain, and brainstem regions that are in control of the autonomic cardiovascular functions (8, 9).

**Figure 1:**
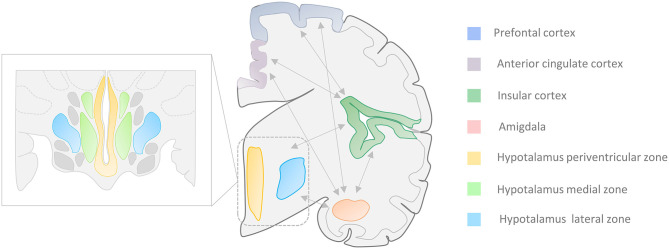
Central Autonomic Network (CAN). Autonomic cardiovascular functions (ACF) are regulated by a complex system called “Central Autonomic Network” that encompasses cortical, midbrain, and brainstem areas. Several portions of the temporal lobe, along with the central nucleus of the amygdala (CeA) and the hypothalamic nuclei, are involved in the ACF regulation. The insular and medial prefrontal cortex, as well as the amygdala, are involved in high-order processing of viscerosensory information and the initiation of integrated autonomic responses. These areas are intimately interconnected with each other as well as with the hypothalamus (periventricular and lateral zone), the anterior cingulate cortex, and brainstem regions. Seizures that arise from the amygdala-hippocampal, cingulate, opercular, anterior frontopolar, and orbitofrontal regions can produce autonomic manifestations that include cardiac arrhythmias, viscerosensory phenomena, vomiting, genitourinary symptoms, and sexual arousal (17–21). *Zoom rectangle*: Functional organization of the hippocampus in the periventricular, medial, and lateral zones.

Additional areas like the central nucleus of the amygdala (CeA) and some hypothalamic regions are also involved in the CAN (10–16). The activation of these structures contributes to the dysregulation of cardiovascular activity as well as the production of arrhythmic and blood pressure changes that are often observed in TLE patients. The two regions are strongly connected to other cortical regions, like the insular cortex (I.C.), the prefrontal cortex (PFC), and the anterior cingulate cortex (ACC), that are also part of the CAN [(8, 9); Figure 1].

Although the mechanisms leading to the development of epilepsy-related autonomic changes are not entirely understood, a current hypothesis postulates that these phenomena result from the progressive alterations, occurring in autonomic centers, that are triggered by repetitive seizure discharges (22). In line with this hypothesis, experimental data indicate that autonomic alterations depend on the pharmacological modulations exerted by specific anti-seizure (ASD) treatments [like carbamazepine (3, 23) and phenytoin (24)] or by the length of the patient epileptic history (25). Furthermore, in drug naïve patients, the autonomic imbalance has been found to be less present in the early stages of the disease but becomes more prominent with the progression of the epileptic process (25). Moreover, chronic TLE can induce structural lesions characterized by the presence of significant neuronal loss and sclerosis in two regions that are involved in the CAN, like the amygdala and the hippocampus (26–28). These structural alterations play a role in the pathophysiology and development of the Sudden Death for Epilepsy (SUDEP) (27–31).

Several studies have suggested the presence of hemispheric lateralization of the autonomic control of the cardiovascular functions. Bradycardic effects have been associated with the stimulation of the left temporal lobe, while tachycardic responses are linked with the right stimulation (32, 33). However, it is still unclear whether transient modifications of the brain activity—like the ones occurring upon interictal epileptic discharges (IED)—influence cardiac functioning in a hemispheric-specific fashion (34). Previous studies have demonstrated that IED affect the occurrence of Heart Rate Variability (HRV) modifications that are observed in focal epilepsy (35). However, little is known about the specific autonomic changes associated with TLE.

The analysis of HRV is a useful tool to evaluate the impairment of cardiac autonomic control (36). The HRV represents the change in the time interval between successive heartbeats (37, 38). HRV provides an index of the parasympathetic nervous system activity (36, 39), whereas possible inferences on sympathetic components have been revised and rejected (20, 36, 40). Some authors (21, 41) have indicated that HRV cannot be employed to infer activities of the sympathetic nervous system as some HRV parameters mostly relate to baroreflex functions. The relationship between HRV and parasympathetic activity has been extensively described (41).

The analysis of HRV has been extensively employed to study changes of the sympathovagal balance that occurs upon physiological responses of healthy subjects as well as in patients affected by cardiac or neurological diseases. It is now well-established that the HRV is reduced in individuals who have epilepsy. The phenomenon is present in newly diagnosed and drug naïve patients (42) and a relevant marker of cardiovascular risks like, for instance, the predisposition to generate ventricular arrhythmias (43–46).

The present study aimed at comparing the interictal changes of HRV that occur in TLE patients differentiated by the right or left location of the epileptic foci. In these two groups, the relationship between interictal epileptiform discharges and modifications of the autonomic cardiac control assessed.

## Methods

### Patient Demographics and Clinical Features

Fifty-two adult patients (24 men and 28 women, mean age 42.9 ± 16.4 years, age range = 20–73 years) affected by TLE with a clear EEG interictal discharges lateralization (right or left) were retrospectively selected from the database of patients who underwent a 21-channel video-electroencephalogram (video-EEG) recording at the Epilepsy Center of the University “G. D'Annunzio” of Chieti-Pescara and the Epilepsy Center of the Catholic University of the Sacred Heart of Rome. Video-EEGs were recorded using a sampling frequency of 256 Hz. During the recording sessions, patients were supine and relaxed. Two neurologists subspecialized in epilepsy made a qualitative evaluation of EEG recordings, highlighted IED, and their specific localization. All the investigated patients exhibited clear lateralization of EEG interictal discharges. Patients with bilateral IED were excluded from the analysis. No ictal events were recorded.

All patients received a diagnosis of TLE based on clinical (43), neurophysiological, and interictal video-EEG, as well as by brain magnetic resonance (MRI) scans performed to determine the epilepsy etiology. The mean disease duration time was 14.9 ± 15.2 (duration range = 1–61 years). All patients were right-handed.

The group was equally divided into a subset of 26 patients presenting with left temporal IED and 26 patients exhibiting right temporal IED. Patients did not exhibit psychiatric comorbidity as assessed by the Symptom checklist-90 (SCL-90) (47). None of the patients were treated with drugs that interfere with the functioning of the Autonomic Nervous System (ANS), including oral contraceptives. None of the investigated patients had a history of heart diseases, endocrine disorders, metabolic deficits, uremia, or any other known disease that could have affected autonomic functions, including sleep-related apnea. Selected patients were no-smoker and had no history of alcohol or drug abuse. No coffee, tea or other energizing drinks, as well as meals, were ingested in the 2 h before EEG recordings. No intense physical activity was reported in the day before EEG. All patients reported regular sleep routines in the 7 days before the recording. Patient responses to pharmacotherapy were analyzed according to the ILAE diagnostic criteria for pharmaco-resistant epilepsy (48). Patients treated with carbamazepine or phenytoin were excluded from the analysis. The clinical and demographic features of the study cohort are summarized in Table 1.

**Table 1:** Demographics and clinical data.

	**Right TLE**	**Left TLE**	
	**(*n* = 26)**	**(*n* = 26)**	
**Age (years)**	40.32 ± 14.32	45.35 ± 18.13	*p* = 0.301
**Disease duration (years)**	16.6 ± 16.072	12.91 ± 14.32	*p* = 0.399
**Seizure types**			*p* = 0.095
Focal	9 (35%)	15 (56%)	
- Focal cognitive seizures	5	11	
- Focal automatism seizures	4	5	
- Focal sensory seizures	0	1	
Focal to bilateral	17 (65%)	11 (44%)	
**Seizure control**			*p* = 0.569
Seizure-free	15 (64%)	17 (65%)	
No seizure-free	11 (44%)	9 (35%)	
**Response to ASD**			*p* = 0.792
Pharmacoresistance	5 (20%)	6 (23%)	
Non-pharmacoresistance	18 (79%)	18 (69%)	
Undefined	3 (11%)	2 (8%)	
**Etiology**			*p* = 0.780
Unknown etiology	11 (44%)	12 (46%)	
Known etiology	15 (60%)	14 (54%)	
*Brain tumor*	3	3	
*Cortical malformation*	7	6	
- Post-traumatic	1	3	
- Ischemic Stroke	2	0	
- Vascular malformation	1	1	
- Multiple Sclerosis	1	0	
- Infectious encephalitis	0	1	

*Demographics, clinical data, and statistical comparisons performed in the right and left TLE patients*.

*TLE, temporal lobe epilepsy; ASD, anti-seizure drugs*.

### EKG Samples and Heart Rate Variability (HRV) Analysis

Bipolar electrocardiogram (EKG) recordings from lead I of a 12-lead EKG were carried out utilizing the EKG channel of the EBN-Neuro EEGNet System (EBN Neuro–Florence Italy). According to guidelines for HRV measurement in epileptic patients (34), we took in consideration EKGs only in patients who were recorded (1) at least 8 h after the last tonic-clonic seizure, (2) at least 1 h after the last known clinical, subclinical electroencephalographic seizure, and (3) at least 1 h before the next seizure. We excluded from the analysis patients who presented a respiration rate above 12 cycles/min (0.2 Hz) during the EKG registration to rule out biases due to individual differences in respiration. EKG data were sampled at a frequency of 256 Hz and exported from the EBN system (EEGNET, Florence, Italy) in the European Data Format (EDF). All data were subsequently processed using dedicated software for HRV analysis (Kubios, HRV software version 2.1, University of Eastern Finland, Kuopio, Finland). The software identified QRS complexes and R peaks using a multiscale wave-let-based peak detection algorithm. Before proceeding with the HRV analysis, all the RRI samples were visually inspected by two trained neurologists to remove any artifacts, extrasystoles, and erroneously detected R waves or insertions of missed R beats. The rate of artifacts that were detected and removed was 5% of all RRI in the EKG recordings.

A short-term recording analysis (49) (time-series length = 5 min) was performed to assess heart rate variations in time and frequency domains as well as non-linear analysis.

The time-domain methods are derived from the beat-to-beat R.R. interval values in the time domain. HRV parameters that measure the variability within the R.R. time intervals in the time-domain assessed in terms of (1) mean R.R. (the mean heart rate in a precise R.R. sequence); (2) SDNN (standard deviation of all R.R. intervals); (3) RMSSD (root mean square of the difference of adjacent R.R. intervals); (4) pNN50 (the percentage of successive R.R. intervals differing more than 50 ms); (5) HRV triangular index (integral of the density of the R.R. interval histogram divided by its height), and (6) TINN (baseline width of the R.R. interval histogram). According to the current literature (50), short-term analysis of SDNN and RMSSD is the most reliable HRV time-domain parameters. SDNN assesses sympathetic and parasympathetically-mediated HRV variations. It should be pointed out that SDNN appears to be more accurate when calculated over 24 h compared to shorter periods, thereby representing the “gold standard” for the medical stratification of cardiac risk. The RMSSD is the primary time-domain measure used to estimate the vagus-mediated changes of HRV (51). Lower RMSSD values correlate with higher scores on a risk inventory for SUDEP (52).

Frequency-domain measurements estimate the distribution of absolute or relative power into four frequency bands. The power spectral density (PSD) of the R.R. series was calculated using parametric methods (based on self-regressive models, AR). PSD was analyzed by calculating the frequency of waves for the different frequency bands. According to the Task Force of the European Society of Cardiology and the North American Society of Pacing and Electrophysiology (1996) (36), H.R. oscillations should be analyzed taking in consideration selected frequencies: Very Low Frequency (VLF, 0–0.04 Hz), Low Frequency (LF, 0.04–0.15 Hz), and High Frequency (HF, 0.15–0.4 Hz). The most common frequency domain parameters include the powers of the bands VLF, LF, HF expressed in absolute (VLF [ms^2^], LF [ms^2^], and HF [ms^2^]) and relative values (VLF%, LF%, and HF%), the normalized power of the LF and HF bands (LF n.u. = LF [ms^2^]/(total power [ms^2^] – VLF [ms^2^]; HF n.u. = HF [ms^2^]/(total power [ms^2^] – VLF [ms^2^]), and the LF/HF ratio.

Spectral analysis allows the discrete analysis of different autonomic components. HF band reports the parasympathetic components, whereas the interpretation of the LF band is controversial (21, 53–59). VLF, LF, and HF bands can be expressed in absolute (ms^2^) or relative (expressed in % or n.u.) units, but absolute values are preferred (59). A recent study (60) investigated the general pattern and timeframe of cardiac autonomic changes that occur upon aging. The study showed that HF as well as LF expressed in absolute units, are decreased by 30–35% with aging. On the contrary, normalized values were affected less (0.8–1.2%).

In line with recent recommendations (17) we present and discuss results of spectral analysis expressed in absolute and normalized units. As recommended by the European Society of Cardiology (ESC) guidelines (36), VLF assessment in a short-term EKG analysis is of dubious value, and its interpretation should be avoided. The LF/HF ratio has been considered as an index of sympathovagal balance. However, this view has been criticized (50). The consensus is now that the precise physiological underpinning of LF/HF is unclear, thereby questioning its predictive value to assess autonomic balance. However, several studies have reported that the index has prognostic value as far as the mortality risk due to cardiovascular or non-cardiovascular causes (61–65).

The non-linear analysis is another alternative way to characterize the variability of heart rate by measuring complex fluctuations of cardiac rhythms. This method allows the definition of the unpredictability of time series resulting from the complexity of the mechanisms that regulate HRV. SD1 and SD2 non-linear parameters can be extrapolated from the Poincaré plot (obtained by plotting every R-R interval against the prior interval and thereby creating a scatter plot). SD1 assesses short-term HRV, correlates with the HF power, and is directly related to RMSSD (66), whereas SD2 investigated short- and long-term HRV and correlates with the LF power. Another useful non-linear parameter is approximate entropy (ApEn), which has been developed to measure the complexity of relatively short time-series. Applied to HRV data, large ApEn values indicate low predictability of fluctuations in successive R.R. intervals (67), whereas small ApEn values indicate signals that are regular and predictable (68). A modified version of ApEn is sample entropy (SampEn), used to assess the complexity of physiological time-series signals (69). SampEn values are interpreted and used like ApEn and can be calculated from shorter time-series (<5 min). Other non-linear parameters like detrended fluctuation analysis (DFA) are designed to analyze time series that span over several hours of data; therefore, their significance in short term analysis is not relevant.

### Statistics

Before comparing the two groups (left vs. right TLE), the normality of distribution of all metric data was tested with the Shapiro-Wilk test. Significance was set at *p* < 0.05. The analysis revealed a non-normal distribution of the data. Metric variables (age, disease duration, MeanRR, SDNN, RMSSD, pNN50, R.R. triangular index, TINN, SD1, SD2, ApEN, SampEN, HF, and LF absolute power, HF and LF normalized unit, HF and LF percentage, LF/HF ratio) were compared employing the one-way analysis of variance with the Mann-Whitney *U*-test. Nominal variables (seizure type, seizure control, response to ASD, etiology) were analyzed and compared between left and right TLE with 2 × 2 contingency tables using the chi-square or Fisher's exact test. Correlations between disease duration and HRV frequency parameters were performed using the Sperman correlation test.

The level of significance was set at *p* < 0.05. Statistical analyses were performed using SYSTAT 12 software (SYSTAT® Software Inc., 2007).

## Results

**Figure 2:**
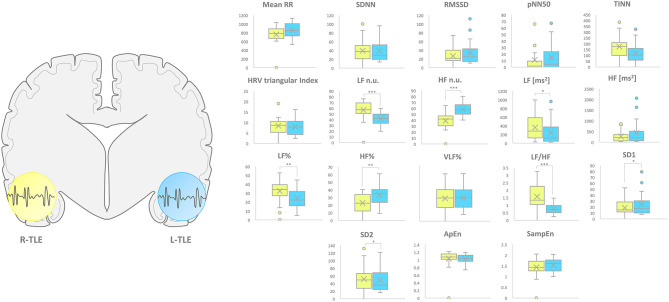
HRV-related parameter comparison in patients with R-TLE and L-TLE. Right (yellow) and left (light blue) graph boxplots depict statistically significant differences in LF n.u. (*p* < 0.01), HF n.u. (*p* < 0.01), LF [ms^2^] (*p* < 0.05), LF% (*p* < 0.0001), HF% (*p* < 0.001), and LF/HF ratio (*p* < 0.0001), SD1 (*p* < 0.05) as well as SD2 (*p* < 0.05) in the two study groups. Mann-Whitney test was employed for statistical analysis. *p* < 0.05 was considered statistically significant. *p < 0.05; **p < 0.001; and ***p < 0.0001.

L-TLE and R-TLE groups did not show any significant difference as far as age (*p* = 0.301), disease duration (*p* = 0.399), seizure control (*p* = 0.569), etiology (*p* = 0.780), and seizure types (*p* = 0.095). The two groups also showed no differences in time-domain parameters (MeanRR, SDNN, RMSSD, pNN50, R.R. triangular index, and TINN). The HRV spectral component analysis indicated a significantly decreased LF/HF ratio in the L-TLE patients when compared to the R-TLE individuals (*p* < 0.0001). Compared to the R-TLE group, L-TLE showed significantly increased HF n.u. (*p* < 0.0001) and HF% (*p* < 0.0001) with decreased LF [ms^2^] (*p* < 0.05), LF n.u. (*p* < 0.001), and LF% (*p* < 0.001). The non-linear analysis showed significantly increased SD1 (*p* < 0.01) and decreased SD2 (*p* < 0.01) in the L-TLE group. No significant differences were observed when comparing ApEN (*p* = 0.133) and SampEN (*p* = 0.570) between the two groups. Time-domain, frequency domain, and non-linear analysis features are summarized in Figure 2. No correlations between disease duration and LF, HF, or LF/HF ratio values were observed.

### Subgroups Analysis

We analyzed and compared HRV parameters in the R-TLE and L-TLE groups taking into account (1) the seizure types (focal seizures vs. focal-to-bilateral tonic-clonic seizures); (2) the response to ASDs (pharmacoresistant vs. no pharmacoresistant); (3) seizure frequency (seizure-free vs. non-seizure-free patients), and (4) the etiology (unknown etiology vs. epilepsy of known etiology).

#### Seizure Type (Focal Seizures vs. Focal-to-bilateral Tonic-Clonic Seizures)

**Figure 3:**
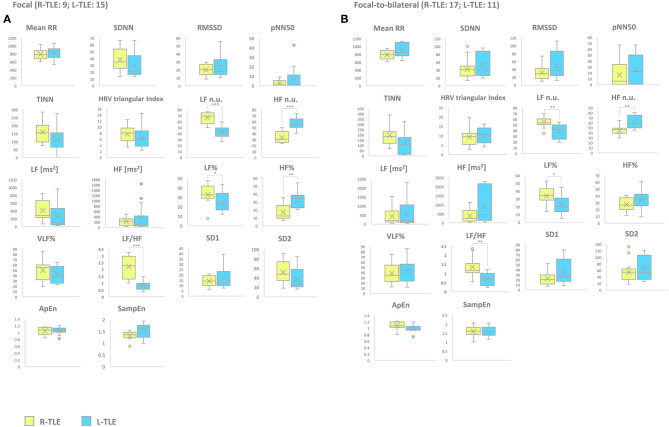
HRV-related parameter comparison in patients with focal or focal-to-bilateral seizures. **(A)** Right (yellow) and left (light blue) graph boxplots depict statistically significant differences in LF n.u. (*p* < 0.0001), HF n.u. (*p* < 0.0001), LF% (*p* < 0.01), HF% (*p* < 0.001), and LF/HF ratio (*p* < 0.0001) of the two study groups of patients suffering from focal seizures. **(B)** Right (yellow) and left (light blue) graph boxplots depict statistically significant differences in LF n.u. (*p* < 0.001), HF n.u. (*p* < 0.001), LF% (*p* < 0.01), and LF/HF ratio (*p* < 0.001) of the two study groups of patients suffering from focal-to-bilateral seizures. Mann-Whitney test was employed for statistical analysis. *p* < 0.05 was considered statistically significant. *p < 0.05; **p < 0.001; and ***p < 0.0001.

Patients with focal seizures in the L-TLE group showed significantly increased HF n.u. (*p* < 0.0001) and HF% (*p* < 0.01) as well as decreased LF n.u. (*p* < 0.0001), LF% (*p* < 0.01), and LF/HF ratio (*p* < 0.001) when compared to homologous R-TLE patients. Patients with focal-to-bilateral tonic-clonic seizures in the L-TLE group showed significantly increased HF n.u. (*p* < 0.001), decreased LF n.u. (*p* < 0.001), LF % (*p* < 0.01), and LF/HF ratio (*p* < 0.001) compared to homologous R-TLE patients. No statistically significant differences were observed between the two groups as far as time-domain parameters (MeanRR, SDNN, RMSSD, pNN50, R.R. triangular index, and TINN) and non-linear analysis (SD1, SD2, ApEn, and SampEn). Time-domain, frequency domain, and non-linear analysis features of the subgroups are summarized in Figure 3.

#### Response to ASDs (Pharmacoresistant vs. Non-pharmacoresistant)

**Figure 4:**
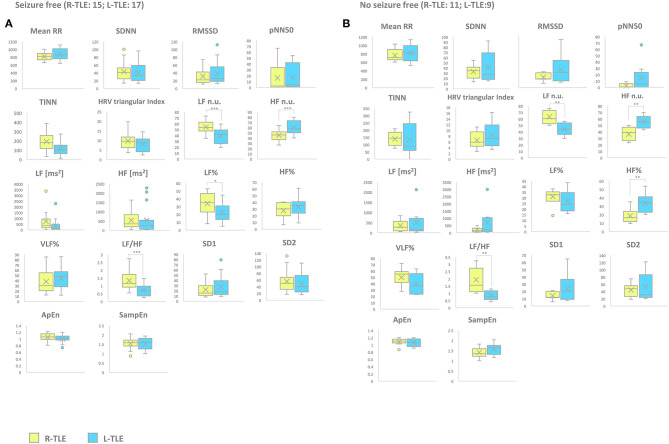
HRV-related parameter comparison in *seizure-free* or *no seizure-free* patients. **(A)** Right (yellow) and left (light blue) graph boxplots depict statistically significant differences in LF n.u. (*p* < 0.0001), HF n.u. (*p* < 0.0001), and LF/HF ratio (*p* < 0.0001) of *seizure-free* TLE patients. **(B)** Right (yellow) and left (light blue) graph boxplots depict statistically significant differences in LF n.u. (*p* < 0.001), HF n.u. (*p* < 0.001), HF% (*p* < 0.001), and LF/HF ratio (*p* < 0.001) *of no seizure-free* TLE patients. Mann-Whitney test was employed for statistical analysis. *p* < 0.05 was considered statistically significant. *p < 0.05; **p < 0.001; and ***p < 0.0001.

Patients with pharmacoresistant epilepsy in the L-TLE group showed significantly increased HF n.u. (*p* < 0.001), HF% (*p* < 0.01) as well as decreased LF n.u. (*p* < 0.001), and LF/HF ratio (*p* < 0.001) compared to homologous R-TLE patients. Patients with non-pharmacoresistant epilepsy in the L-TLE group showed significantly increased HF n.u. (*p* < 0.0001), HF% (*p* < 0.01), decreased LF n.u. (*p* < 0.0001), LF% (*p* < 0.01), and LF/HF ratio (*p* < 0.0001) when compared to matched R-TLE patients. No statistically significant differences were observed between the two subgroups as far as time-domain parameters (MeanRR, SDNN, RMSSD, pNN50, R.R. triangular index, and TINN) and non-linear analysis (SD1, SD2, ApEn, and SampEn). Time-domain, frequency domain, and non-linear analysis features are summarized in Figure 4.

#### Seizure Frequency (Seizure-Free vs. No Seizure-Free Patients)

**Figure 5:**
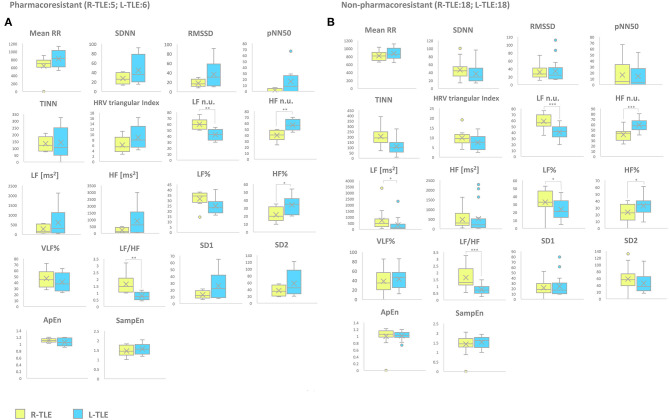
HRV-related parameter comparison in patients with pharmacoresistant or non-pharmacoresistant epilepsy. **(A)** Right (yellow) and left (light blue) graph boxplots depict statistically significant differences in LF n.u. (*p* < 0.001), HF n.u. (*p* < 0.001), HF% (*p* < 0.05), and LF/HF ratio (*p* < 0.001) of pharmacoresistant TLE patients. **(B)** Right (yellow) and left (light blue) graph boxplots depict statistically significant differences in LF n.u. (*p* < 0.0001), HF n.u. (*p* < 0.0001), LF [ms^2^] (*p* < 0.05), LF% (*p* < 0.01), HF% (*p* < 0.01), and LF/HF ratio (*p* < 0.001) of non-pharmacoresistant TLE patients. Mann-Whitney test was employed for statistical analysis. *p* < 0.05 was considered statistically significant. *p < 0.05; **p < 0.001; and ***p < 0.0001.

Seizure-free patients in the L-TLE group showed significantly increased HF n.u. (*p* < 0.0001), decreased LF n.u. (*p* < 0.0001), LF% (*p* < 0.01), and LF/HF ratio (*p* < 0.0001) compared to homologous R-TLE patients. No seizure-free patients in the L-TLE group showed significantly increased HF n.u. (*p* < 0.001), HF% (*p* < 0.001), decreased LF n.u. (*p* < 0.001), and LF/HF ratio (*p* < 0.001) when compared to homologous R-TLE patients. No statistically significant differences were observed between the two subgroups as far as time-domain parameters (MeanRR, SDNN, RMSSD, pNN50, R.R. triangular index, and TINN) and non-linear analysis (SD1, SD2, ApEn, and SampEn). Time-domain, frequency domain, and non-linear analysis features are summarized in Figure 5.

#### Etiology (Unknown Etiology vs. Known Etiology)

**Figure 6:**
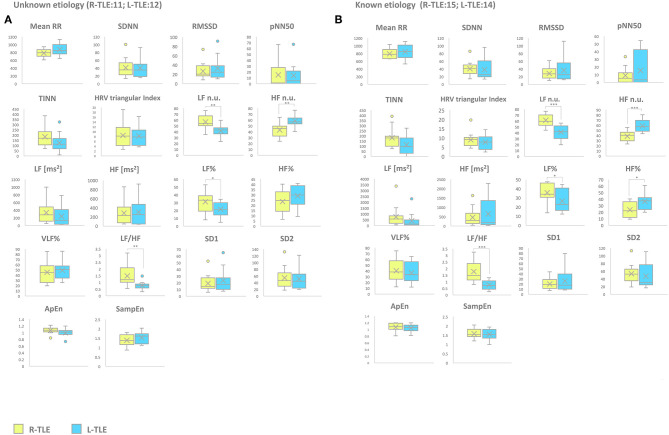
HRV-related parameter comparison in patients with epilepsy with unknown or known etiology. **(A)** Right (yellow) and left (light blue) graph boxplots depict statistically significant differences in LF n.u. (*p* < 0.001), HF n.u. (*p* < 0.001), LF% (*p* < 0.05), and LF/HF ratio (*p* < 0.001) of unknown etiology TLE patients. **(B)** Right (yellow) and left (light blue) graph boxplots depict statistically significant differences in LF n.u. (*p* < 0.001), HF n.u. (*p* < 0.0001), LF% (*p* < 0.05), HF% (*p* < 0.05), and LF/HF ratio (*p* < 0.0001) of known etiology TLE patients. Mann-Whitney test was employed for statistical analysis. *p* < 0.05 was considered statistically significant. *p < 0.05; **p < 0.001; and ***p < 0.0001.

Patients with epilepsy with unknown etiology in the L-TLE group showed significantly increased HF. n.u. (*p* < 0.001), decreased LF n.u. (*p* < 0.001), LF% (*p* < 0.01), and LF/HF ratio (*p* < 0.001) when compared to matched R-TLE patients. Patients with epilepsy with known etiology in the L-TLE group showed significantly increased HF n.u. (*p* < 0.0001) and HF% (*p* < 0.01), decreased LF n.u. (*p* < 0.0001), LF% (*p* < 0.01), and LF/HF ratio (*p* < 0.0001) when compared to homologous R-TLE patients. No statistically significant differences were observed in the two subgroups as far as time-domain parameters (MeanRR, SDNN, RMSSD, pNN50, R.R. triangular index, and TINN) and non-linear analysis (SD1, SD2, ApEn, and SampEn). Time-domain, frequency domain, and non-linear analysis features are summarized in Figure 6.

## Discussion

The present study aimed at comparing the interictal changes of HRV in TLE patients who were differentiated by the right or left location of the epileptic foci. R-TLE and L-TLE subjects were investigated to assess the relationship between interictal epileptiform discharges and modifications of the autonomic cardiac control. Our data show that R-TLE patients exhibit a reduced parasympathetic tone, as indicated by the presence of HF reductions. These patients also exhibit a higher risk of mortality as assessed by the LF/HF parameter.

Autonomic dysregulation and HRV variations are important cardiovascular risk factors as these alterations underlie a potentially harmful decrease of the parasympathetic tone.

The reduced parasympathetic tone may predispose to a “pro-arrhythmic” condition (44, 46, 70), which, in turn, may lead to an increased risk of SUDEP. The quantification of HRV is an established method to assess the parasympathetic activity of the ANS. The standard recording period of ≤ 5 min (short-term analysis) is now recommended by the Taskforce of the European Society of Cardiology as a valid procedure for the assessment of HRV components (36).

Clinical data concerning the possibility of a lateralized control of autonomic functions in epileptic patients are controversial. Although HRV features associated with ictal events have been extensively described (70–72), little is known about the role of IED changes in the cardiac autonomic regulation of patients with epilepsy. By definition, interictal epileptic discharges are brief spikes or sharp waves that are not associated with clinical symptoms. Despite their brief nature, IED play an essential role in the development of some epilepsy-related comorbidities, including cognitive decline (73, 74) and hormonal changes (75). However, the role of IED in the development of cardiac alterations is not well-understood. Compared to healthy controls, patients with epilepsy exhibit a higher incidence of subtle abnormalities in HRV as well as abnormalities in the cardiac response to physiological stimuli (3, 4, 6, 76).

In patients affected by generalized seizures, the presence of HRV alterations that are related to IED were initially described by Faustmann and Ganz (76). These authors have indicated that patients with normal interictal EEG exhibit HRV that are similar to healthy controls. HRV modifications may be driven by ASD. Carbamazepine (3, 23) and phenytoin (24), in particular, have been shown to promote HRV modifications even though the role of carbamazepine is controversial (77). Other studies, especially those investigating drug-free, naïve, patients (42), reported that the role of ASD in the modification of HRV is marginal, thereby stressing the importance of the central control exerted by the cortex on the activity of the ANS.

Our results are in line with data indicating the presence of an asymmetrical autonomic innervation of the heart as well as the lateralization of the cardiac autonomic output in the brainstem. These data are thereby suggesting a different contribution of the two hemispheres in the control of the heart rate. In that context, preclinical and clinical data have demonstrated that lesions of the right hemisphere produce an increased sympathetic tone (33, 78). Furthermore, the stimulation of the left insula has been shown to induce a bradycardic response, whereas tachycardia and pressor responses are more elicited from the stimulation of the right insula (32). Studies based on the pharmacological inactivation of both hemispheres, obtained through the intracarotid injection of amobarbital, have produced conflicting results (79–81). One study (79), albeit robust in terms of the size of the enrolled patients, suffers from methodological issues related to the length of the HRV evaluation (i.e., ultra-short lasting analysis) and the presence of anticonvulsant treatment as 65 of the 73 enrolled patients have been treated with carbamazepine or phenytoin.

Additional evidence supporting the notion of lateralization of the control of the cardio-autonomic functions comes from studies employing functional Magnetic Resonance Imaging (fMRI). fMRI data related to the cortical control of spontaneous and arousal-induced fluctuations in the amplitude of skin conductance responses (SCR) support the presence of a sympathetic activity that is mainly controlled by the right hemisphere (82). These findings are also confirmed by electrophysiological studies performed on patients with side-specific hemispheric lesions that indicated a decreased galvanic skin response in patients with right-hemisphere lesions. In contrast, increased responses were found in subjects with lesions of the left hemisphere (83, 84).

Studies employing positron emission tomography to investigate hemispheric-specific increases of blood flow showed increased perfusion in the right insula of healthy patients undergoing physical activity or exposure to mental stress. In contrast, increased blood flow occurred in the left insula of subjects performing non-strenuous tasks (85).

## Conclusions

Our data provide experimental evidence to support the notion of lateralized cortical control of the cardiac autonomic functions in TLE patients. In particular, our findings suggest that epileptic patients with an L-TLE focus exhibit a lower risk of developing cardiac dysfunctions independently of the disease duration. Given the well-known correlations between the presence of HRV modifications and the occurrence of SUDEP, it can be inferred that patients with L-TLE may be less susceptible to develop SUDEP. A recent study (86) attempted to define the incidence of clinically relevant arrhythmias in refractory focal epilepsy and assessed the predicting value of postictal arrhythmias as risk markers for SUDEP. The study investigated people with refractory epilepsy (both TLE and non-TLE) who were implanted with a loop recorder and followed for 2 years. The study found no clinically relevant arrhythmias during the follow-up. It is conceivable that the inclusion of non-TLE patients in the study could have blurred the results, thereby open the possibility that findings may be different when taking into consideration only TLE patients and when employing HRV alterations as selection criteria.

We acknowledge some limitations of our study. Given the small number of patients in the subgroup analysis, we were not able to investigate the impact of every specific ASDs in the modification of HRV features. However, although the role of the most recent ASDs in the modulation of HRV remains to be defined, our data indicate that, independently of specific ASDs, R-TLE patients exhibit a reduced vagal tone. Further studies will be needed to understand the impact that specific ASDs may have on HRV modifications as well as to assess the role of pharmaco-resistance in producing worse cardioautonomic balance. Finally, our results indicate that it would be important to investigate, with long-term EKG monitoring, whether R-TLE patients show a higher risk of developing arrhythmias.

## Data Availability Statement

The datasets generated for this study are available on request to the corresponding author.

## Ethics Statement

Ethical review and approval was not required for the study on human participants in accordance with the local legislation and institutional requirements. The patients/participants provided their written informed consent to participate in this study.

## Author Contributions

FD, GE, MO, and CV contributed to the conception and design of the study. GE organized the database. CV performed the statistical analysis. FD, VF, GE, and SS wrote the manuscript and supervised all the data. MF evaluated EKG data. All authors contributed to manuscript revisions, read, and approved the submitted version.

## Conflict of Interest

The authors declare that the research was conducted in the absence of any commercial or financial relationships that could be construed as a potential conflict of interest.
